# Avirulence Effector Discovery in a Plant Galling and Plant Parasitic Arthropod, the Hessian Fly (*Mayetiola destructor*)

**DOI:** 10.1371/journal.pone.0100958

**Published:** 2014-06-25

**Authors:** Rajat Aggarwal, Subhashree Subramanyam, Chaoyang Zhao, Ming-Shun Chen, Marion O. Harris, Jeff J. Stuart

**Affiliations:** 1 Department of Entomology, Purdue University, West Lafayette, Indiana, United States of America; 2 Department of Agronomy, Purdue University, West Lafayette, Indiana, United States of America; 3 USDA-ARS and Department of Entomology, Kansas State University, Manhattan, Kansas, United States of America; 4 Department of Entomology, North Dakota State University, Fargo, North Dakota, United States of America; INRA, France

## Abstract

Highly specialized obligate plant-parasites exist within several groups of arthropods (insects and mites). Many of these are important pests, but the molecular basis of their parasitism and its evolution are poorly understood. One hypothesis is that plant parasitic arthropods use effector proteins to defeat basal plant immunity and modulate plant growth. Because avirulence (*Avr*) gene discovery is a reliable method of effector identification, we tested this hypothesis using high-resolution molecular genetic mapping of an *Avr* gene (*vH13*) in the Hessian fly (HF, *Mayetiola destructor*), an important gall midge pest of wheat (*Triticum* spp.). Chromosome walking resolved the position of *vH13*, and revealed alleles that determine whether HF larvae are virulent (survive) or avirulent (die) on wheat seedlings carrying the wheat *H13* resistance gene. Association mapping found three independent insertions in *vH13* that appear to be responsible for *H13*-virulence in field populations. We observed *vH13* transcription in *H13*-avirulent larvae and the salivary glands of *H13*-avirulent larvae, but not in *H13*-virulent larvae. RNA-interference-knockdown of *vH13* transcripts allowed some *H13*-avirulent larvae to escape *H13*-directed resistance. *vH13* is the first *Avr* gene identified in an arthropod. It encodes a small modular protein with no sequence similarities to other proteins in GenBank. These data clearly support the hypothesis that an effector-based strategy has evolved in multiple lineages of plant parasites, including arthropods.

## Introduction

Many gene-for-gene interactions are manifestations of the biological interplay that occurs between plant resistance proteins and plant pathogen effector proteins [1]–[5]. Plant pathogens use their effector proteins to defeat basal plant immunity and modify plant cell biochemistry and development [6]. The resistant plant host counters this attack using resistance (*R*) gene encoded proteins that detect specific effectors or effector activity [1], [4], [5], [7]. The resulting R-protein/effector interaction elicits a plant resistance response called effector-triggered immunity (ETI) [2], which restricts the proliferation of the pathogen. Not all effector proteins elicit ETI, but those that do are called Avirulence effectors (Avr effectors), and the genes that encode Avr effectors are called *Avirulence* (*Avr*) genes [8]. *Avr* gene cloning was instrumental in achieving this understanding, and the first method used to identify pathogen effectors [9]. It remains a reliable approach to effector discovery [10].

Like most plant pathogens, large numbers of plant-feeding arthropods (mites and insects) have intimate, highly specialized and obligatory relationships with their plant hosts. It also appears that many of these arthropods use an effector-based strategy of plant attack [11]–[13]. Evidence supporting this hypothesis comes from an examination of both the plant and the arthropod. The plant *R* gene *Mi* is an important example [14], [15]. *Mi* confers resistance to the potato aphid (*Macrosiphum euphorbiae*), white flies (*Bemisia tabaci*) and root knot nematodes (*Meloidogyn* ssp.). Like many pathogen resistance proteins, the Mi protein contains nucleotide binding (NB) and leucine rich repeat (LRR) motifs [16], [17], suggesting that it interacts with aphid and white fly effectors. Genetic data in a variety of plants also supports the existence of many other cultivar-specific *R* genes that guard against insect and mite effectors [18]–[20]. On the arthropod side of the interaction, plant physiological responses to aphid saliva have been attributed to effectors [11], [21], and both effector and candidate effector proteins have been identified in a few arthropod species [11], [13], [22]–[25]. Gene-for-gene interactions have also been documented between two gall midges, the Hessian fly (*Mayetiola destructor*) and the Asian rice gall midge (*Orseolia oryzae*) and their respective plant hosts, wheat (*Triticum* spp.) and rice (*Oryza sativa*) [12], [26], [27]. However, an arthropod Avr effector has yet to be identified.

**Figure 1:**
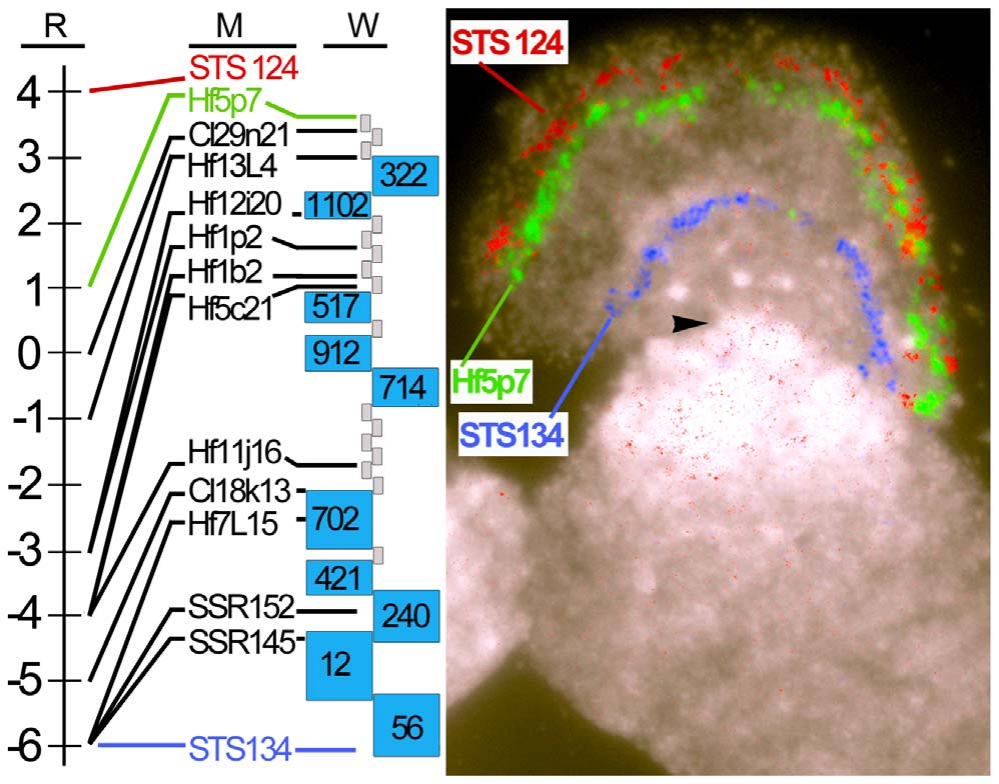
Mapping *vH13*. (A) The scale shows the number of recombinant individuals in the BC mapping population (n = 106) at markers (M) identified in a chromosome walk (W). The walk proceeded from marker 134 towards marker 124 and was composed of BACs (grey boxes) and FPC-based BAC contigs (blue boxes). (B) Fluorescence *in situ* positions of markers 124, Hf5p7 and 134 on the short arm of HF polytene chromosome X2. The arrowhead indicates the position of the X2 centromere.

In this study, we used a map-based approach to clone an arthropod *Avr* gene from the Hessian fly (HF), a plant-galling insect and an important insect pest of wheat (*Triticum* spp.). Previous investigations indicated that the wheat *R* gene *H13* has an *Avr* gene cognate that would be an excellent candidate for a map-based cloning effort [28], [29]. *H13* itself is a simply inherited dominant *R* gene located in a cluster of genes encoding NB and LRR motifs on wheat chromosome 6DS [30], [31]. Its *Avr* cognate (*vH13*) was previously mapped between two molecular markers (124 and 134) on the short arm of HF chromosome X2 (Figure 1). *vH13* segregates as a simply inherited genetic factor that determines whether HF larvae will survive or die on *H13*-wheat seedlings (Figure S1) [28]. Recombination rates (87-kb/cM) near marker 124 suggested that map-based gene identification might be possible in that region [29]. As genetic traits, *H13*-resistance in wheat, and *H13*-avirulence (larval death) and *H13*-virulence (larval survival) in the HF are unmistakable and 100% penetrant (Figure S1) [28]. *H13*-avirulent larvae are unable to modulate *H13*-plant development [32], but *H13*-virulent larvae create nutritive tissue at the feeding site, and permanently stunt *H13*-seedling development [33].

Here, we identify mutations (insertions) in a single HF gene that are perfectly associated with the ability of the insect to avoid *H13*-directed ETI. These mutations were genetically and physically mapped in two structured mapping populations and four different unstructured field-collected populations. We found that the candidate gene carrying these mutations encodes a protein that has features in common with many effectors: it is a small modular protein bearing a predicted signal peptide that has no sequence similarity to other proteins in GenBank. It is expressed in *H13*-avirulent first-instar larvae and *H13*-avirulent larval salivary glands, but not in *H13*-virulent larvae. We also found that RNA-interference-based knockdown of this candidate gene's expression can transform *H13*-avirulent larvae into *H13*-virulent larvae. We therefore conclude that this candidate is *vH13*, the first *Avr* gene identified in an arthropod.

## Materials and Methods

### Plant and Insect Materials

USDA-ARS investigators Dr. R. Shukle, Dr. B. J. Schemerhorn and S. Cambron generously provided wheat seed and HF material used in this investigation. Insect rearing and experimental matings were performed using near isogenic wheat lines Newton (fully HF susceptible) and Molly (*H13*-resistant) [34]. HF strains used in this investigation have been described previously [29], [35]. All strains were maintained as families of individual females on caged pots of wheat seedlings at 20±2°C as previously described [35]. Field collections of the HF were made at Pointe Coupee Parish Louisiana, Baldwin Co. Alabama, Spalding Co. Georgia, and Orangeburg Co. South Carolina and shipped to S. Cambron at Purdue. These insects were maintained in diapause at 4°C. All of the females used in this investigation produced either all-female or all-male offspring.

### Genetic Mapping

We used both structured and non-structured HF populations to perform molecular genetic mapping. Two structured mapping populations were generated from separate crosses between individual *H13*-virulent males and two sister *H13*-avirulent females, one female-producing and one male-producing (Figure S2). Subsequently, F_1_ males and females collected from each population were separately inter-mated to produce two different F_2_ populations. F_2_ males were separately collected from both populations and genotyped as hemizygous *H13*-virulent (*v/-*) or hemizygous *H13*-avirulent (*A/-*) in testcrosses as described below (Figure S2). All of the F_2_ males in one structured population (named BC) were collected and genotyped. From the other population (named RIL), some of the F_2_ males were genotyped while others were mated to F_2_ females to produce an F_3_ population. Continued inbreeding maintained the RIL population to the F_6_ generation. RIL males were collected and genotyped from the F_3_ to the F_6_ generations. Non-structured, association mapping was performed by genotyping individual males collected from the four field populations as described below.

To genotype individual males collected from both structured and non-structured populations as hemizygous *H13*-virulent (*v/-*) and hemizygous *H13*-avirulent (*A/-*), we performed separate testcrosses with homozygous *H13*-virulent (*v/v*) individual virgin females (Figure S2). Single *H13*-virulent males (*v/-*) testcrossed to individual *H13-*virulent (*v*/*v*) females produced *H13*-virulent female (*v*/*v*) offspring. Single *H13*-avirulent males (*A/-*) testcrossed to individual *H13*-virulent (*v*/*v*) females produced avirulent (*v*/*A*) female offspring. Testcrosses that produced male offspring were uninformative; testcross males were always *H13*-virulent (*v*/-) because they were always hemizygous for their mother's X2 chromosome.

### Chromosome walking

To identify bacterial artificial chromosomes (BACs) containing marker 134, we screened three different HF BAC libraries (available upon request) as previously described [29]. To continue the walk, PCR-amplified ^32^P-labelled probes were prepared based on BAC-end sequence (GenBank Trace Archive TI numbers 2136865139-2136875614 and 2136877165-2136888504 as part of BioProject PRJNA63389), and these were used to screen the same BAC libraries. FPC-based BAC contigs facilitated the walk [36], and the continuity of the walk was tested using FISH [35]. The BACs identified in each step of the walk and the primers used to both generate BAC-end probes and identify the DNA polymorphisms that were used as molecular markers during the walk are presented in Table S1.

### Gene annotation

BAC Hf5p7 sequence (deposited at GenBank, Accession No. HQ540429) was annotated using GenScan [37], and FGENESH [38] software. Artemis software [39] was then used to perform manual annotation based on the results of the GenScan and FGENESH predictions.

### Real-Time PCR

Quantitative real-time reverse transcription-PCR (qRT-PCR) was performed using an ABI PRISM Fast 7500 Detector and the SYBR Green I dye-based detection system (Applied Biosystems, Foster City, CA) as described previously [40]. PCR was performed in a final reaction volume of 10 µl using the following cycles: 50°C for 2 min, 95°C for 10 min, 40 cycles of 95°C for 15 s and 60°C for 30 s. Target-specific primers were designed using Primer Express Software Version 3.0 (Applied Biosystems). The Relative Standard Curve Method (User Bulletin 2: ABI PRISM 7000 Sequence Detection System) was used to quantify gene expression. Relative expression analyses were performed using a HF *Ubiquitin* gene transcript (UBQ; GenBank DQ674274.1) as the internal reference. Relative expression of candidate gene 13 (*vH13*) was determined using 4 biological replicates each with three technical replicates. Data are depicted as per cent expression of *vH13* transcripts normalized to UBQ, in the treated larval samples relative to the control larval samples. The forward UBQ primer sequence in these experiments was 5′-CCCCTGCGAAAATTGATGA-3′ and reverse was 5′-AACCGCACTACTTGCATCGAA- 3′ and the *vH13* forward primer and reverse primer sequences were 5′-GGTTGCTTTTATAGTTTTGGCCAT-3′ and 5′-AAATTGTCGATCACATGCATCATA-3′.

### RNAi

Cloned cDNA in the vector pCRII-TOPO (Invitrogen) was used as template for the amplification of *vH13* cDNA using both the *vH13* specific forward primer described above with a 5′-T7-promoter sequence extension and a different *vH13* reverse primer (5′-CTTCTCCTTCTTGGCTCTC-3′) with 5′-T7-promoter sequence extensions. The product of this reaction was gel-purified using the Qiaex II gel extraction kit (Qiagen), and 0.2 µg of the product was used as template for an *in vitro* transcription reaction using T7 MEGAscript Kit (Ambion) performed according to the manufacturer's recommendations. Avirulent HF first-instar larvae were collected in water as they hatched from eggs deposited on wheat leaves. The larvae were then incubated in water mixed with 10 mM Octopamine and either cowpea weevil (*Callosobruchus maculatus*) alpha amylase gene dsRNA, or *vH13* dsRNA. Treated larvae were then placed, five at a time, on the developing third leaf of separate wheat seedlings in the 2^nd^-leaf growth stage and permitted to move down and feed at the base of the plants. The plants were checked daily for stunting, and they were dissected and examined for living and dead larvae 20 days after infestation.

## Results

A chromosome walk was initiated using an HF BAC (Mde37L4) containing *vH13*-linked marker 134 (Figure 1). The walk progressed distally on the short arm of the chromosome, towards *vH13* and marker 124. BAC contigs that had been previously constructed using high-information content fingerprinting and FPC software facilitated this effort [36], [41]. Fluorescence *in situ* hybridization (FISH) of BACs to the polytene chromosomes of the HF was used to test the fidelity of the walk (Figure 1, Table S1). F_2_ males (n = 106) collected from a structured mapping population (BC) were genotyped as *H13*-avirulent and *H13*-virulent (Figure S2) and used to genetically position BAC-end sequences relative to *vH13* (Figure 1, Table S1).

**Figure 2:**
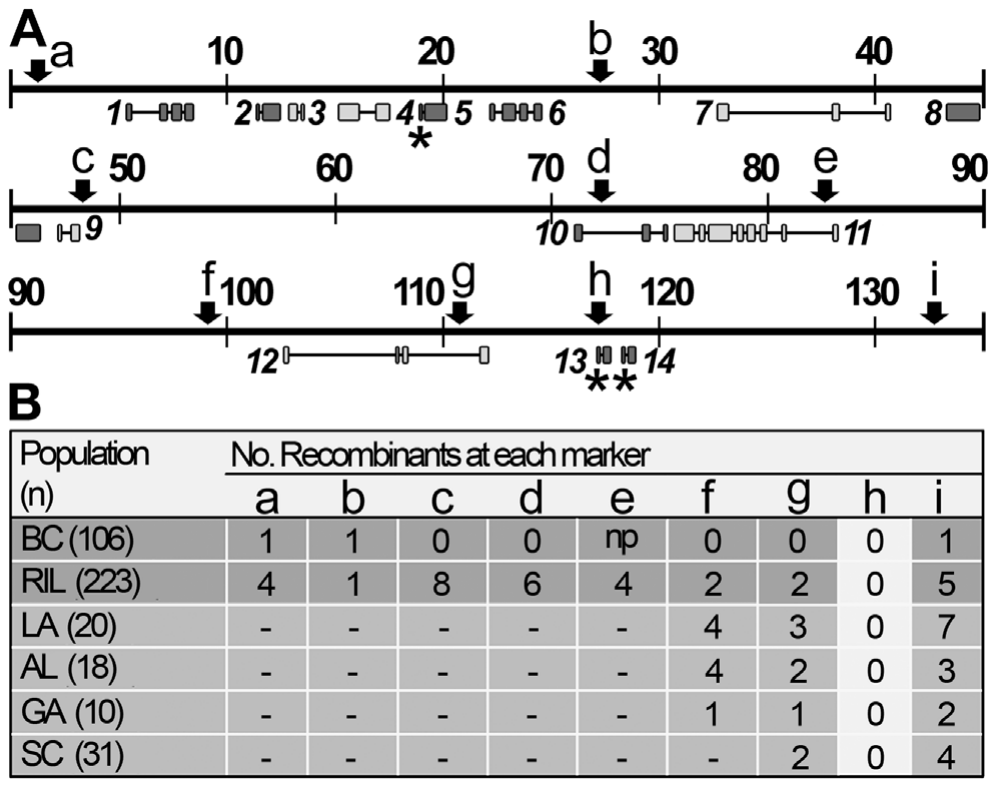
Mapping *vH13* within BAC Hf5p7. (A) Map showing the positions of the molecular markers (a-i) that were used to refine the position of *vH13* on BAC Hf5p7 (scale = kb). Predicted genes are shown below the map. Genes transcribed from left-to-right are colored dark grey and genes transcribed from right-to-left are colored light grey. Asterisks indicate genes encoding predicted signal peptides. (B) Table showing the numbers of recombinant individuals within structured mapping populations (BC and RIL) and field populations (LA, AL, GA and SC) at each of the markers (a-i) shown in A.

Genetic analysis performed during the chromosome walk indicated that the likely position of *vH13* was between the ends of a single HF BAC (Hf5p7; Figure 1). BAC Hf5p7 was then sequenced and annotated (GenBank Acc. No. HQ540429, Table S2). This permitted us to both develop additional PCR-based markers within the HF5p7 sequence (Figure 2AB, Table S3) and make candidate *Avr* gene predictions (Figure 2A, Table S2). Using only the BC mapping data, *vH13* mapped between DNA polymorphisms at position 28-kb and 134-kb within the BAC Hf5p7 sequence (Figure 2AB, positions b and i). Only eight putative genes (candidate genes 7 through 14) were in this region (Figure 2A, Table S2). Two of these genes (candidates 13 and 14) had attributes characteristic of known *Avr* genes: they were relatively small (1.4 kb and 1.7 kb respectively) and appeared to encode signal peptides (SignalP, *P* = 1.0) [42]. Candidate 13 had 2 predicted exons encoding 116 amino acids. Candidate 14 had 3 predicted exons encoding 106 amino acids. The predicted amino acid sequences of candidate genes 13 and 14 had only 13% similarity, and neither candidate had significant sequence identities with other genes in GenBank (BLASTX and BLASTN≥e = 1.0).

**Figure 3:**
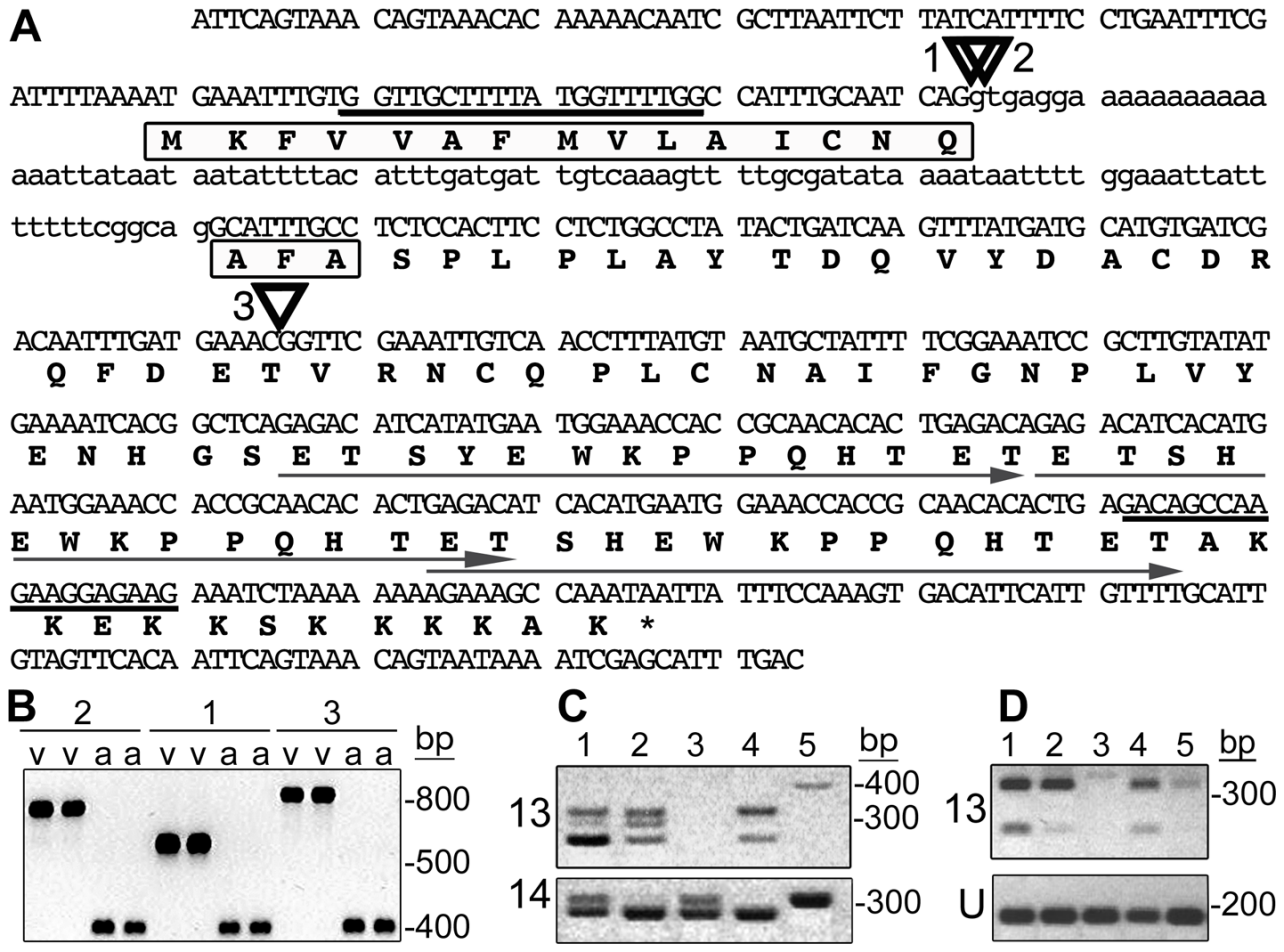
*vH13* candidate gene 13 structure and expression in *H13*-virulent and avirulent strains. (A) *H13*-avirulent genomic DNA sequence of *vH13* candidate-13 showing exons (capital letters), intron (lower case letters), PCR primer-targeted sites (bold underlining), the positions of virulence-associated insertions (triangles 1, 2 and 3) and the predicted amino acid sequence (bold letters). The predicted signal peptide is boxed and the three imperfect direct repeats are underlined with arrows. (B) Candidate-13 fragments amplified using genomic DNA template extracted from *H13*-virulent (v) and *H13*-avirulent (a) individuals. *H13*-virulence associated sequences correspond to the insertions (1, 2 and 3) shown in panel A. For an explanation of the band lengths, see Figure S3. (C) Candidate-13 (13) and candidate-14 (14) transcripts amplified using total RNA extracted from pools of first-instar larvae (KS-GP, lane 1; IN-L, lane 2; vH13, lane 3 and IN-vH9, lane 4). Only candidate-14 sequence was amplified using the RNA extracted from the pool of *H13*-virulent first-instar (vH13, lane 3). Genomic DNA extracted from a single KS-GP larva was amplified as a control (lane 5). (D) Amplification of candidate-13 (13) and HF-ubiquitin (U) gene sequences using total RNA extracted from pools of *H13*-avirulent first-instars (lane 1), second-instars (lane 2), third-instars (lane 3), first-instar salivary glands (lane 4), and the carcases of first-instar larvae after salivary gland removal (lane 5).

To refine the position of *vH13* in BAC Hf5p7, we developed a second structured mapping population (RIL) and genotyped males (n = 223) selected from the F_3_ through the F_6_ generations of that population (Figure S2). *vH13*-recombinant males were identified in this population at eight of the nine Hf5p7 sequence markers (Figure 2AB, markers a-g and i). However, no recombination was observed between *vH13* and the polymorphism at position 117-kb (Figure 2AB, marker h). That polymorphism resided within the sequence of one of the best candidates: candidate gene 13. Sequencing this polymorphism revealed the presence of a 4.7-kb insertion at the putative exon-intron junction of candidate gene 13 (Figure 3AB, insertion 1; Figure S3). The insertion consisted of 149-bp inverted repeats flanking 4,474 bp encoding a peptide with significant sequence similarity to a hypothetical *Hydra magnipapillata* protein (BLASTP, e = 3^−37^). A direct repeat (2 bp) of target DNA flanked the insertion, suggesting that it was the remnant of a transposable element. The insertion was present in all RIL *H13*-virulent males, but absent in all RIL *H13*-avirulent males. Thus, its position and distribution were consistent with the possibility that it caused *H13*-virulence by disrupting candidate-13 function.

To test the association of candidate gene 13 with *H13*-virulence further, we performed association mapping using *H13*-virulent and *H13*-avirulent males collected from field populations in Louisiana (LA), Alabama (AL), Georgia (GA), and South Carolina (SC). Again, we discovered that insertions in candidate gene 13 near position 117-kb in the BAC Hf5p7 sequence were perfectly associated with *H13*-virulence, while flanking polymorphisms, 6-kb and 16-kb distant, recombined (Figure 2AB, Figure 3AB, Figure S3). The same 4.7-kb insertion segregating in the RIL mapping population was present in all AL and GA field-collected *H13*-virulent HFs. A smaller insertion (254 bp), present near the exon-intron junction of candidate 13, was present in all SC *H13*-virulent HFs (Figure 3AB, insertion 2; Figure S3). A third insertion (461 bp), located in the coding region of the second putative exon, was present in all LA *H13*-virulent HFs (Figure 3AB, insertion 3; Figure S3). The latter insertion was also present in all *H13*-virulent F_2_ males in the BC population and accounted for the indel observed in that population at BAC Hf5p7 position 117-kb (Figure 2AB, marker h). No insertions of any type were ever observed in *H13*-avirulent HFs in any of the structured or non-structured populations. Because the three insertions had no significant sequence similarities to each other (BLAST 1e<1.0) [43], and each was inserted at a different position, it appears that the *H13*-virulence associated insertions arose independently (Figure S3). The genetic data from each mapping and field-collected population placed *vH13* within 22 kb of the BAC Hf5p7 DNA sequence between markers at positions 111-kb and 133-kb (Figure 2, markers g and i). The only candidate genes residing within this sequence, candidates 13 and 14, encode proteins with predicted signal peptides. We failed to identify any *H13*-associated polymorphisms within candidate gene 14. Therefore, the position and segregation of the *H13*-virulence associated insertions clearly suggested that candidate gene 13 is *vH13*.

To explore this possibility further, we examined the transcription of both candidates 13 and 14 and the predicted proteins they encode. Full-length candidate-*13* cDNA sequence (Figure S4) confirmed that the gene is composed of only two exons, where the first exon encodes a predicted signal peptide and the second encodes the predicted mature protein (Figure 3A). Therefore, its gene structure resembles the majority of the candidate HF effectors originally discovered as transcripts in the HF salivary gland [23]. Reverse transcription PCR (RT-PCR) revealed evidence of candidate-13 transcription in *H13*-avirulent larvae and *H13*-avirulent first instar salivary glands (Figure 3CD). However, no evidence of transcription was observed in *H13*-virulent first-instar larvae (Figure 3C). This pattern of transcription was perfectly congruent with the expression of an *Avr* gene whose product elicits *H13*-directed resistance, an ETI that kills avirulent first-instar larvae. In comparison, candidate gene 14 was transcribed in both *H13*-virulent and -avirulent first-instar larvae (Figure 3C). Candidate-13 transcripts of three different lengths were amplified from RNA extracted from avirulent larvae. The longest transcript encoded a 116-amino acid protein (Figure 3A) that has no sequence similarity to other proteins in GenBank (BLASTP e≥0.004 and TBLASTX 1e≥0.016) [44]. However, its small, modular structure resembled cytoplasmic oomycete and fungal effectors [3], [45], as well as the candidate effectors discovered in the HF salivary gland transcriptome [46]. A signal peptide was predicted with cleavage between the 18^th^ and 19^th^ amino acids of the protein (SignalP, *P* = 1.0) [42]. The protein also contains an imperfect direct repeat of 14×3 amino acids between residues 63 and 103 (Figure 3A). Interestingly, three *H13*-avirulence associated alleles were identified, each encoding one to three of these imperfect repeats (Figure S5). These alleles accounted for the three different transcripts amplified from pools of *H13*-avirulent, first-instar, larval RNA (Figure 3CD). The downy mildew (*Hyaloperonospora arabidopsidis*) ATR13 effector has signal cleavage sites and imperfect, direct, amino acid repeats in similar positions [47]. In addition, both *ATR13* and *vH13* candidate-gene 13 have alleles encoding different numbers of imperfect repeats. Like ATR13, the number of repeats would appear to have no predicted affect on candidate-13's ability to elicit ETI because alleles encoding all three variants are present in populations that are purely *H13*-avirulent (Figure 2B, Figure 3C). Nevertheless, the existence of these alleles suggests that candidate-13 is experiencing diversifying selection, an attribute that is also consistent with a role as an effector protein [3], [23], [47].

**Figure 4:**
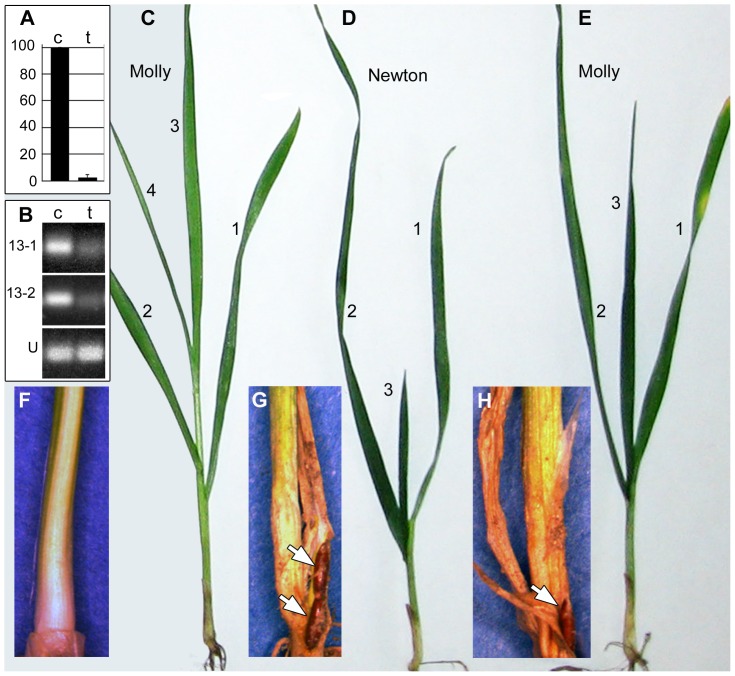
*vH13* knockdown allows *H13*-avirulent larvae to escape *H13*-directed ETI. Pools of 100 *H13*-avirulent neonate larvae were soaked in 0.5 mg/ml of either sham-, or *vH13*-dsRNA for 48 h. (A) Percent transcription of *vH13* in *vH13*-dsRNA-treated larvae (t) relative to sham-treated larvae (c) as measured using qRT-PCR. (B) Amplification of the *vH13* transcript (13-1 and 13-2) and the ubiquitin transcript (U) from RNA samples extracted from sham-treated (c) and *vH13*-treated (t) larvae after 35 cycles of RT-PCR. Ubiquitin transcript amplification was performed using the same RNA used in 13-1. (C-H) Similarly treated larvae were transferred, five per plant, to *H13*-resistant (Molly), or susceptible (Newton) near-isogenic wheat seedlings. Plants shown 12 days after infestation (C, D, and E) have their leaves numbered. Stunted plants (D and E) were darker green than unstunted plants (C) and never developed a fourth leaf. HF pupae (arrows) were visible on stunted plants 20 days after infestation (F, G and H). Sham-treated larvae failed to stunt (C) and survive (F) on Molly, but did stunt (D) and survive (G) on Newton. Some candidate-gene-13-dsRNA-treated larvae also stunted (E) and survived (H) on Molly.

Taken together, the congruence of candidate 13 gene structure with that of an effector, the presence of insertions in candidate gene 13 in virulent individuals and the lack of candidate-gene-13 expression in *H13*-virulent larvae all strongly suggested that this candidate is an *Avr* gene. Therefore, we attempted to test this hypothesis further using a functional assay based on RNA-interference (RNAi). This method was modified after the approach used to knockdown nematode genes [48], and to our knowledge, it is the first instance in which the procedure was applied to the HF. To target candidate 13, we used a dsRNA molecule that had no significant similarities to any other HF gene (BLASTN e≥0.28) [44] (Figure S4) in the HF genome database (HessianflyBLASTdb) [49]. We were therefore confident that we would not observe off-target effects. Pools of 100 neonate *H13*-avirulent larvae were exposed for 48 h in aqueous solutions of candidate-gene-13 dsRNA (0.5 mg/ml). Although we could not measure knockdown in individual larvae, we did discern that the treatment reduced the relative expression of the gene in pools of larvae to 2.5±2.2% of control pools of larvae soaked in sham dsRNA, *Callosobruchus maculates* alpha amylase gene, GenBank Acc. No. FK668918 (Figure 4AB). This suggested that the treatment might achieve a knockdown that would be sufficient to allow some *H13*-avirulent larvae to escape *H13*-directed resistance. We then transferred similarly treated larvae to seedlings of near isogenic *H13*-resistant ‘Molly’ and fully susceptible ‘Newton’ wheat lines [34] (Figure 4C-H). The treatments starved the larvae for 48 h, which we presumed would weaken the ability of the larvae to move to an appropriate feeding site, induce the formation of nutrient tissue, and survive. In an attempt to compensate for this, we transferred 5 larvae to each seedling. This permitted averages of 1.6±1.0 larvae treated with sham dsRNA and 1.5±1.1 larvae treated with candidate-13 dsRNA to survive on susceptible Newton plants 20 days after infestation. Eighty-six percent (43/50) of the susceptible Newton plants infested with larvae treated with sham dsRNA and 80% (40/50) of the Newton plants infested with larvae treated with candidate-13 dsRNA were fully stunted and had surviving larvae (Figure 4DG). No (0/118) *H13*-resistant Molly plants infested with larvae treated with sham-dsRNA were either stunted or had living larvae (Figure 4CF). However, 5.3% (9/168) of the *H13*-plants infested with candidate-13 dsRNA treated larvae were permanently stunted and had living larvae 20 days after infestation (Figure 4EH). Because Molly (*H13*) plants were, and always have been, 100% effective in killing avirulent first-instar larvae in this and all preceding investigations [28], we attributed the escape of these larvae to RNAi-mediated candidate-13-knockdown. This result clearly indicated that candidate 13 is *Avr* gene *vH13*. It also suggests that it may be possible to use RNAi to study the effects other putative HF effectors have in the modulation of wheat seedling development and gall formation.

## Discussion

Several lines of evidence suggest that candidate gene 13 is *Avr* gene *vH13*. First, molecular mapping resolved the position of *vH13* to only two candidate genes, and although the genomic architecture of both genes resembled other putative HF effector-encoding genes [23], further analysis clearly indicated that candidate 13 was *vH13* and that candidate 14 was not. Spontaneous DNA insertions in candidate gene 13 were perfectly associated with the segregation of *H13*-virulence in six independent HF populations, but there were no allelic differences associated with candidate gene 14. Similarly, the absence of candidate gene 13 transcripts in virulent larvae was perfectly consistent with *Avr* gene loss-of-function, whereas the presence of candidate gene 14 transcripts in *H13*-virulent larvae was not. Moreover, and consistent with this observation, RNAi-based knockdown of candidate-gene-13 expression was associated with escape from *H13*-directed ETI. Taken together, we conclude that candidate gene 13 is an effector-encoding *Avr* gene, and by extension, that this insect uses an effector-based strategy to modulate the development of its host.

The HF belongs to the large gall midge family (Cecidomyiidae) in the order Diptera [50], which in terms of species diversity, is the most successful group of plant-galling insects [51]–[53]. Most gall midge species have complicated life cycles that make them difficult to rear. In addition, their hosts typically lack the genetic resources of wheat. Thus, the vast majority of the interactions that occur between thousands of gall midge species and their hosts lack the genetic tractability of the HF-wheat interaction. The same is true of thousands of other plant parasitic arthropod species. This accounts for the very limited number of examples of plant-arthropod gene-for-gene interactions, even as evidence for the existence of arthropod effectors grows. Conversely, this also suggests that the genetic tractability of the wheat-HF interaction should be fully exploited. Over 30 HF *R* genes have been discovered in wheat germplasm [54]. HF avirulence to five of these *R* genes has already been shown to segregate like different *Avr* genes on HF chromosomes [12], [55]. Therefore, we hope that *vH13* is only the first of several arthropod effector-encoding *Avr* genes that will be identified in the HF.

Hundreds of putative HF effectors, called secreted salivary gland proteins (SSGPs), have been identified in the first-instar HF larval salivary gland transcriptome [56]. Although *vH13* has structural features in common with these, it lacks any significant sequence similarity (BLASTN e≥0.28) [44]. Nevertheless, we believe that common structural features and salivary gland expression indicate that some of the SSGPs may correspond to other HF *Avr* genes. Like other effectors and immune-related genes, putative HF effectors exhibit sequence patterns that are consistent with high diversifying selection for functional adaptation; the non-coding segments of some of the related SSGPs have greater similarities than segments encoding the mature proteins [23]. Such sequence diversity also makes it difficult to determine how *vH13* and the SSGP gene sequences arose. One possibility is that the genes have expanded and diversified after an ancient horizontal transfer. Phylogenetic evidence suggesting that gall midge herbivory arose from mycetophagous ancestors is certainly consistent with this hypothesis [57], as is the existence of maternally transmitted bacterial HF symbionts [58]. However, because effectors diversify so rapidly, this hypothesis may prove difficult to test.

## Conclusions

High-resolution molecular genetic mapping and association mapping identified mutations that allow the HF to survive on wheat plants carrying the *H13* resistance gene. These mutations consist of insertions that reside within a small candidate *Avr* gene composed of two exons; the first exon appears to encode a secretion signal and the second appears to encode a mature protein. The presence of the mutations is perfectly associated with the absence of an associated transcript in *H13*-virulent HF larvae. RNAi-knockdown of the candidate gene's expression rescued a small number of *H13*-avirulent larvae on *H13*-resistant wheat plants. We therefore conclude that this candidate gene is an effector-encoding *Avr* gene (*vH13*) and the first *Avr* gene identified in an insect.

## Supporting Information

Figure S1 **Phenotypes associated with the wheat-HF gene-for-gene interaction.** (A) *H13*-resistant (R) and susceptible (S) wheat seedlings 20 days after infestation. The susceptible plant is stunted, showing no growth after the emergence of the third leaf. (B) The outer leaves of an *H13*-wheat seedling have been removed to reveal many small, reddish, dead *H13*-avirulent first-instar larvae at the base of the resistant plant 8 days after infestation (bar = 0.5 mm). (C) The outer leaves of a stunted susceptible wheat seedling were removed to reveal living, *H13*-virulent, second-instar larva near the base of the plant 8 days after infestation (bar = 1 mm). The larvae in both (B) and (C) are facing down.
(TIF)

Figure S2 **Generation and genotyping males within structured mapping populations.** (A) Females produce either all-female or all-male families. Males transmit only their maternally inherited chromosomes, and are haploid for the X2 chromosome. Sister P_1_ females, homozygous for *H13*-avirulence (A), are mated to the same *H13*-virulent (v) P_1_ male. These matings produce heterozygous F_1_-female and hemizygous F_1_-male families. Sister, F_1_ females are then mated to a single F_1_ male to produce F_2_ families. The F_2_, and subsequent generations, are then allowed to freely inter-mate and reproduce in isolation (light grey boxes) on susceptible wheat. Males are collected from the F_2_ and subsequent generations (dark grey circles) for genotyping. (B) Testcrosses are performed to genotype males as *H13*-avirulent (Avr) or *H13*-virulent (vir). Males are mated individually to single homozygous virulent females. The females are then caged separately on pots containing susceptible (S) and *H13*-resistant (R) seedlings in opposite halves of the pot. Avirulent males produce female TC families (v/A) that fail to stunt R seedlings. Virulent males produce female TC families (v/v) that stunt R seedlings.
(TIF)

Figure S3 **Genomic DNA sequences of** ***H13*** **-virulent associated insertions.** The insertions are numbered according to their position in the gene as shown in Figure 3A. (A) Insertion-1, present in the RIL, AL, and GA populations. (B) Insertion-2, present in the SC population. (C) Insertion-3, present in the BC and LA populations. Grey highlight = exons; lower case lettering = intron; purple lettering = first copy of a 42-bp (14-amino acid) imperfectly repeated sequence; italicized and underlined lettering = start translation site; italicized and bolded lettering = stop translation site; yellow highlighting = primer target sequences; blue lettering = insertion; black bold lettering = duplicated sequence; blue, bold, and underlined lettering = inverted repeat.
(DOCX)

Figure S4 ***vH13*** **candidate gene 13 cDNA sequence.** Purple lettering indicates one copy of a sequence that is followed by two imperfect copies. Underlined sequence corresponds to the dsRNA used to knockdown *vH13* expression.
(DOCX)

Figure S5 **Genomic DNA sequences of** ***H13*** **-avirulent candidate-13 alleles.** (A) Allele with three imperfect repeats. (B) Allele with two imperfect repeats. (C) Allele with one copy and no repeats. Colors and lettering are as described in Figure S2.
(DOCX)

Table S1 *vH13* chromosome walk progression.
(DOCX)

Table S2 Predicted genes in the HF BAC Hf5p7 sequence.
(DOCX)

Table S3 Marker and primer positions in the HF BAC Hf5p7 sequence.
(DOCX)
