# Supplementary figures and images for: Avirulence Effector Discovery in a Plant Galling and Plant Parasitic Arthropod, the Hessian Fly (Mayetiola destructor)
Source: PLoS One. 2014 Jun 25;9(6):e100958. doi: 10.1371/journal.pone.0100958 (PMC4071006; doi:10.1371/journal.pone.0100958)

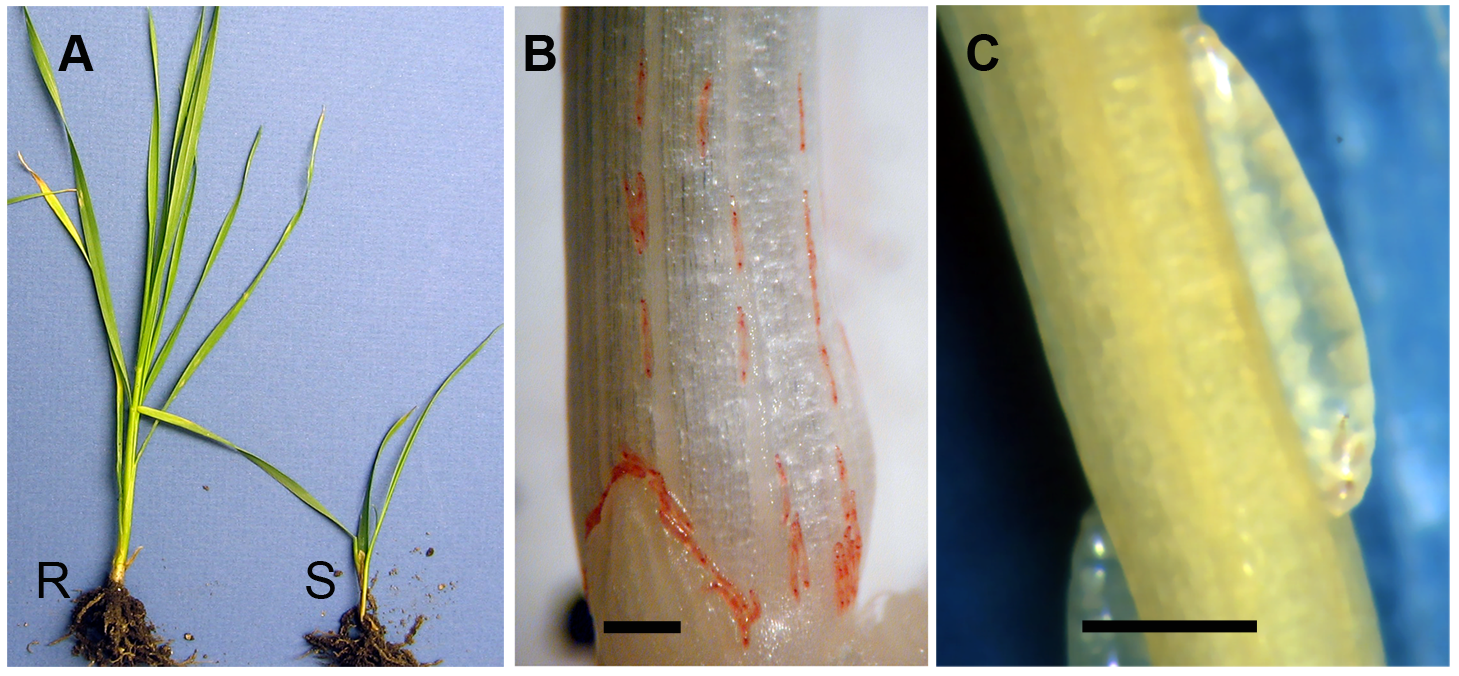

Supplement: Figure S1 — Phenotypes associated with the wheat-HF gene-for-gene interaction. (A) H13-resistant (R) and susceptible (S) wheat seedlings 20 days after infestation. The susceptible plant is stunted, showing no growth after the emergence of the third leaf. (B) The outer leaves of an H13-wheat seedling have been removed to reveal many small, reddish, dead H13-avirulent first-instar larvae at the base of the resistant plant 8 days after infestation (bar = 0.5 mm). (C) The outer leaves of a stunted susceptible wheat seedling were removed to reveal living, H13-virulent, second-instar larva near the base of the plant 8 days after infestation (bar = 1 mm). The larvae in both (B) and (C) are facing down. (TIF) [file pone.0100958.s001.tif]

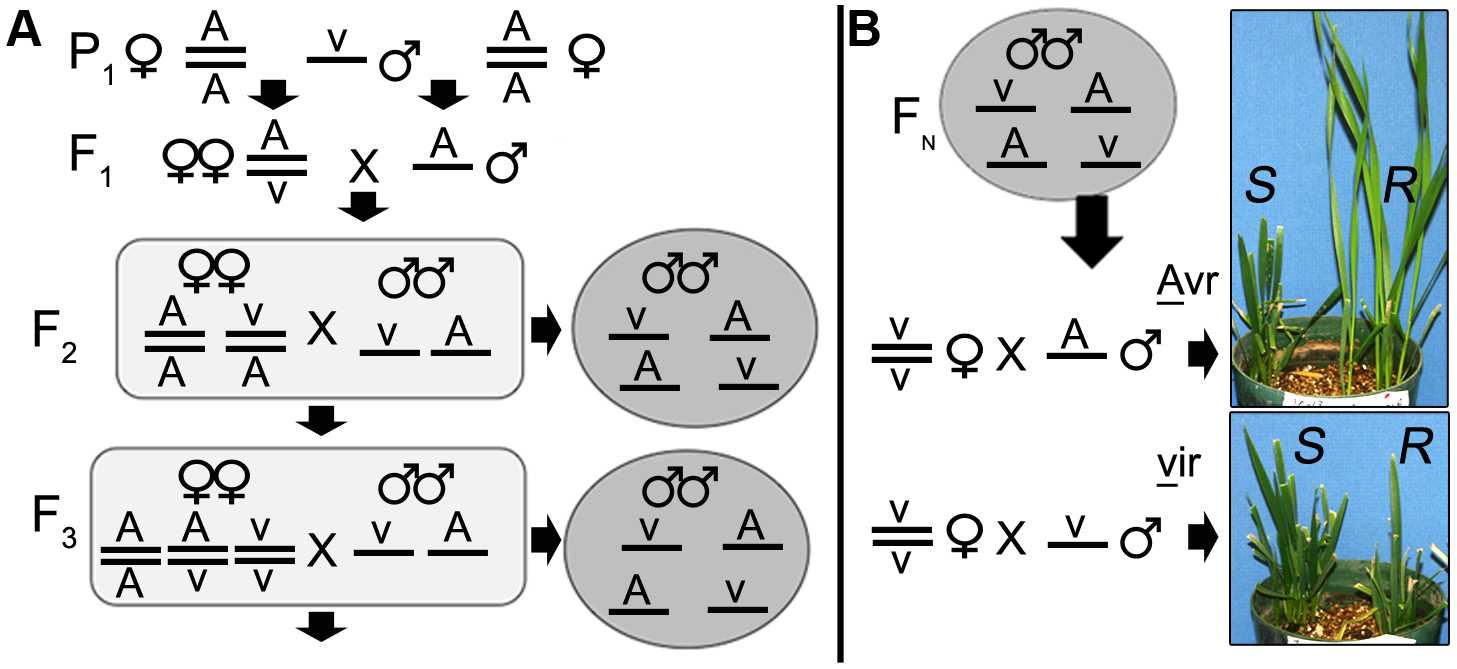

Supplement: Figure S2 — Generation and genotyping males within structured mapping populations. (A) Females produce either all-female or all-male families. Males transmit only their maternally inherited chromosomes, and are haploid for the X2 chromosome. Sister P1 females, homozygous for H13-avirulence (A), are mated to the same H13-virulent (v) P1 male. These matings produce heterozygous F1-female and hemizygous F1-male families. Sister, F1 females are then mated to a single F1 male to produce F2 families. The F2, and subsequent generations, are then allowed to freely inter-mate and reproduce in isolation (light grey boxes) on susceptible wheat. Males are collected from the F2 and subsequent generations (dark grey circles) for genotyping. (B) Testcrosses are performed to genotype males as H13-avirulent (Avr) or H13-virulent (vir). Males are mated individually to single homozygous virulent females. The females are then caged separately on pots containing susceptible (S) and H13-resistant (R) seedlings in opposite halves of the pot. Avirulent males produce female TC families (v/A) that fail to stunt R seedlings. Virulent males produce female TC families (v/v) that stunt R seedlings. (TIF) [file pone.0100958.s002.tif]
