# Supplementary material for: Avirulence Effector Discovery in a Plant Galling and Plant Parasitic Arthropod, the Hessian Fly (Mayetiola destructor)
Source: PLoS One. 2014 Jun 25;9(6):e100958. doi: 10.1371/journal.pone.0100958 (PMC4071006; doi:10.1371/journal.pone.0100958)
Supplement: Figure S3 — Genomic DNA sequences of H13 -virulent associated insertions. The insertions are numbered according to their position in the gene as shown in Figure 3A. (A) Insertion-1, present in the RIL, AL, and GA populations. (B) Insertion-2, present in the SC population. (C) Insertion-3, present in the BC and LA populations. Grey highlight = exons; lower case lettering = intron; purple lettering = first copy of a 42-bp (14-amino acid) imperfectly repeated sequence; italicized and underlined lettering = start translation site; italicized and bolded lettering = stop translation site; yellow highlighting = primer target sequences; blue lettering = insertion; black bold lettering = duplicated sequence; blue, bold, and underlined lettering = inverted repeat. (DOCX) [file pone.0100958.s003.docx]

A GCATCGTAAA CAAAAGCAAA ATAAAATAAT TTTAGAATTC GATCAAATTT CGTTGTTTTT AAATAAGCTT AGTTCACGAA GGGGGTACGT GATCTTTGAA TTGAAGTCAA TAACAACATC CGTTTTGTTT GAATTGTTAT TGACTTCAAA TTATGCATGA TATTATTTAT TCTTATTTTT TTTATTACTT AATAAATGTT AAATTCATTT TGAAAGCTAT TTTTGTTTCA TTATTCAAAT CGAACAGGAT GCCGAAACGA AATATATTAT CATCAATATA AAATGCGTGT CAATAACAAA CAAAATATAC AATTTGCATA TTAATATTTC GAAATTGAAC AAAAATTTTT TAATATAAAT AGATCACCTG AAAGCTGACA TTTTATTCAG TAAACAGTAA ACACAAAAAC AATCGCTTAA TTCTTATCAT TTTCCTGAAT TTCGATTTTA AA*ATG*AAATT TGTGGTTGCT TTTATGGTTT TGGCCATTTG CAATC**AGCTC TCTCATAAAT TTTTAGCAAA TCGGTATGAC TATAATAGCA ACGTATTACT TTACCCACAG TTTTCATATG TGTAGGTAAA GTAATGCGGC CGTATGCTAT GGACGTATGT ATTCACATAC AGCATGCGTC GAACGCATTT GTGTTA**CGCC TTGTTTCTGT ACTTAGCGTA TGCTTCCGTG AGACTTAATA CATTTATACG GTATAAACCG TAATGCGGTT ACCGTATGCT TTATATCGAA TTGTTTTAAA AAAACACTGG TTTCGGTAAT ACAACGGTCC AAGTAGCAAT GAACATTTGA AATTGGATTC AAATAAGATA ATTTCTTGCA AAAATGATTA TTTTCGTCAA AACTCGGATT CAATGTGCGT TTAGGTTTAT TAAAATCGAA GACGAGCAGT TGAATGATAT TGTTGGTTTC GTCAAAAAGG GTAAATATTT GGTTGATGTT TTGAAGATTC AAATAATTCA TGCTTTTAAG ATTTTCATTT CAATTAGCTC TCGCGGCACA TTCAATCAGT TATCAAGACT CTCTCAAATT CAGAATAATC GATAAGGATG GTACACCTAT TTCTGGACAA GATTTCGATG GTTTAATAAG ACAGTATTAT AAATCCAATT GCTTTTTTAT TGAAATTGTC ATTATAAATG GTACCAACGG CAATAGAGAT TTGACGGAGG TATATTTTGT ATTCTCAGTT AGATTTGAAT GGAAATAATG ATTAAATTTT ATCAGCGCCT ACAGAATCAT ATCAAAAACA CCAATAATGA GTTCATAATT GTTGAACATG AACGTTTACG ATATCTTTCG AAAAGTACAC GTAAAACTTT GATTAACGTT TTGATGAGTT TCATACGGGA CGAATTTAAT AATCAACCGT CTAAGCCTGA GATAGCCGAT TTGTGTCTTG CTGCAATTAA AATGTTTCCC AGTTTAAAAG CAGAAAATAG TATTCATGGT GGAATCGTAA GCAATTTTTT GACATTTTGG TGTTTTTGGC TACATACTCA AATAATTGAA TGTTTTCATC ACAGGATCAT TTATATCATC CCGAGAGAAA GTCTGGATTC CTCTATGAAA AACTAAGCAA GCTAGGCACG CAGGCAAATG CTGTTCAAAA TGCGGTTTCT GAAGAAGAGA AATCCAGAAT TATCACATTT TTTACTAATT GCAAATTGCC AAATGACAGA AAAGAAATCG AAAAAGAAAT GAAAGAATCT GTACATATGC GGCACAAACT TCTTTACAAT TCAGCATCAA ATTTCCCAAA AGTGTCCCCA TTTTATGTTG TGAATCCGGA ATTAGTTAGT TGAATTTCAA AACGAGTACA TTTTCATAAT AATCTAACTA TTTCAAATCG TTGCAGATAC TTTTTGATTT TGAGCTCATG TATGAGAAAA TCGATAAAAA CAGTTTGATT GAAATTTGGC CACGAATCAA AAATATTCCT ACGCAAAAAT TTAAATTGGC ATTATTGCCT CAAGAGTCGA GCCATCCAAT GATTGACAAT GTTTTATTGT TTTTGAAACT ATTCCCATCG GGTCGAACTG CATTCAAAAC ATCAATGAAT TCATTTATCA AAATCTCGCA GGATTCAAAA ATGGATCCGG CAATTATGGT AGATAAAAGT ATCGACAGTC CACATATCAT CGCTATTTTA TCAGAAATAT CATCGGATGT TAAATATTAC ATATATTTAG AGCACCGATT TATTTCAGTG AGTACATCTT CAATTGATTT ATAATAATAA AATGATGTGT GGAGATAGCT AAACCCGCAT TTTTTTCTCT TTTAAGCTGC CGATGGATTG GGATTTTATC TTTGTTTTCG ACCTATTTAT CAAAATTCAT TTGGTTTTCA ACATTCAATA TCATTCGAGT TTAAAGCAAT TCATGATATT TTTTGAGAAT TTCGCATACA ATATCAATGA AAAATCTCAT TCTCCAGCAG CACATGAGAC ATGTATGCAA ATAAAGTACA GCAAAATGAA ATTGCGAATA CGGAACAGTA ACTTTCAAGT TACGCGATTC AATTTTTTCA ATCGTTGAAT TTTCATTCCT TTAAAGACAT AATTTGAATC CGCTATCGAC CGAGAAAAAC ATATTTCACA ATATTTTCAT TTATTTAATC AAATTTATTT AATTGTTTGA AAGGACAATA TTTAACATTT ATATTGACAG ATAAAAAATA ATAAAAATAA TTTGAATTTT TAAACAAATG GCAAATCCAA AGTTGATGCG GCTACGTGAA TTTTACCGTT AACATGAATT TTTTCGAAGG GTTTTTTTGT CTGGAGTTTT TCAAACGCAA TAATCTCATG TGAATCAGAA TAGGTGTCAA TTTCAATACT ATGTAAAATC TTATTGATGT TGACAATTTT TCGCTCTATA CACACGAAGT AGAATTCTAT CTCGGAGGAT AAGACATGGT CAATTTCAAA TATCTTATTT TCTTTCAAAA TAATCAACCC TTTTTCATAT CGATAACCGT TGAATTTCAA AAACTTTAGT TTCGTTAATG AGCACAAATT TAGTGAAATA TTTTCTGAAT AAATATCAAC GAATGACTTA AATTCAACAC AATCCTTCAA GCAACAATTA TTCTTTGAAA TTTCAATCTG GTCCGAATAG CATGATTTGG TTTCACATGT GGATTCTTGA TGCCGGTTTG CGAGGGTTTT TGTAATATTG ACGAAGTTTT GAGTTCTGCG CGCTAAATCG GTGAATACTT TATGTTTTGC TTCTATTCTC ATCATTGTAC TGTGAATCGG TGGACCCATT GCTCTGATCA CTGTCGGATA GTGTGAAAGT ATGTGATGCT TGAATATAAA ACGATCATTG AAAACACGTT TCAGTTCGAT TAGGTGTTCG TTAATCAGAT CAGTCAATTT TAAAATGTCC ACTTCCGTGA TTCTCGTTGA ATATATTATT TGCATTATTG AGAGTAAAGT TGTTAAAGGC TTCCATGCAG TTATGAGTTT CTCTTGATAT TCCATCATGA TAAACGGTAA ATTTTGTATC AAACAATGTA GCTGAATTGA ACTTTGGCCC AAATGGGTTT TTTTCATTCC GATTATTGAT GGTTTGTTTT TATTACTGAG CTGGCCATAA TCAAAATCTC TTACTTTTTT AATGATATCA ATTGTTTTTA TACCAGTTTT GTCACAATAG TTTATGAAAA GTTCGATAAA ATGTGGCACA ACCCCTTCTT GCACATCATG CATTAAATCA ACTGACCATT TTTGGAGAAT ATTAAAATAT TTCAATAGTT GAAAACACAT GGCTTCACAA TACCCAAAGT TTCTTTTGGG TTCGAAATGT TTTGATTTAA AATGTTCTCA ATTAGACTAT TGTACACGTT CAATGTTCTT ATTTTGTCTG GGCACTCAAT AGTAAGATTT CGTCGATCAT CAATACTGCT GGTGCAAATT CGGCATGAAT ATTGTGCATT GAAACATTTA ACGAAACCTG ATACCGTGTT CGCACCCAAA TTATCAGCGA GAATTTGAAT TAAACTCCCA TTCAGTTTTA ATTGGTCGTG TATCATGATC CCTTCGGTCT CTAAGATGTT GAGTTCATTT ACAATGATAT TCGAGATATG TTCGATGTGA TCGGTTTCAA TGTCATTATT TGTCGTTTTA CACAATGCAA CTAAATGGAT ATTTTTTAGA CGTGATCGAT ATTGTTTTGG TATATTCCGA ATTTGAAAAT AAATTCCCGT CATTTTATGA ATTTTCACTT TGCTTTTTAT GGGACAACAA ACCTCAAAAT CATCTATTCC TAGCTGTATT TGAATAGAAA GTGGTGGATT GAAAACATCC AATTCCTTGA ATCTTTTACC GCAACAAAAA TCAACATAAC AACCATCAAC ACATTCATGT TTATTCATAT TGTAATCATT GTACATTGCA CGAAAATCTT TGTCCGAAAA CAATGCAGTT AATGTTTCTA AAATCGAAAT ATATTGGAAG GATTCTTGAA CCAATTTATA ATCCGGCAAA TCACAGCTCG ATTTCTGTAA AACCCATTTG AAACCTAATG CCATTTCTTT TGGTTGAACA TATTTTGAAA TTGATTTTAA TTGTTTTTGT CGTTTGTATC TTGTTGAGTT CTCATCAATT TTTCGGTAAA CATATTCGTG GATATTGGAT ACGATTTTTT TAGTTGGTTC ATTCATTGGA TTTGTCGATA AAAAATTATT TAAGTGTTCA TTGCACGCTT TTTTCAATTC TTCTGATATC ATTTTGAACA AATCATATAT TTTGTTCTCT TTGTCATGTT CAAGAAACAT AGAGGTAATC TCACTTTTCA CTGTTTGCAA TGCATCGTCG AGATTCTTAT AGGTTTTGTT CTGAATGGAA ATCTGTTCAA TGCCGGATTC GATCACTTCA ATTTCAAAAA TCTCATCACT CTGAACTGCT GATTCGATAT TATTCTCAAA AATTACTTCA TCACCACGAA GTTCGAAAAT GAATTCATTA TCAAATGGCA TCTTTCAATG TTTATTTAAA TTGGCGATAT TGTTACAGAT ATGGATTTTT ATTTTTAAAT TCAATCATAG CAATCAAACG ACAAAATTAT GTGTAGCAGA GCGGTGCCGC ACAAACATAC ATTTAAAGCC **TAACACACAA GCGTTCGACG CATGCTGTAT GTGAATACAT ACTTCCATAG CATACGCCCG CATTACTTTA CCTACACATA TGAAAACTGT GGGTAAAGTA ATACGTTGCT ATTATAGTCA TACCGATTTG CTAAAAATTT ATGAGAGAGA G**gtgaggaaa aaaaaaaaaa attataataa tattttacat ttgatgattg tcaaagtttt gcgatataaa ataattttgg aaattatttt tttcggcagG CATTTGCCTC TCCACTTCCT CTGGCCTATA CTGATCAAGT TTATGATGCA TGTGATCGAC AATTTGATGA AACGGTTCGA AATTGTCAAC CTTTATGTAA TGCTATTTTC GGAAATCCGC TTGTATATGA AAATCACGGC TCAGAGACAT CATATGAATG GAAACCACCG CAACACACTG AGACAGAGAC ATTACATGAA TGGAAACCAC CGCAACACAC TGAGACATCA CATGAATGGA AACCACCGCA ACACACGGAG ACAGCCAAGA AGGAGAAGAA ATCTAAAAAA AAGAAAGCCA AA***TAA***TTATT TCCAAAGTGA CATTCATTGT TTTGCATTGT AGTTCACAAT AAAATCGAGC ATTTGACAGA ACGGTAAACA ATTTGAAAAG AACGACGTGC AAAATGTAAA ATTCTACCTA TATCCATCTC TTTTCTCACT TAAAACCAAA TGAATTTCTT TCACATTGGT TCAATTTTGC ACGTCGTTCC TTAAAAATTG C

B GCATCGCAAA CAAAAGCAAA ATAAAATAAT TTTAGAATTT GATCAAATTT CGTTGTTTTT AAATAAGCTT AGTTCACGAA GGGGGTACGT GATCTTTGAA TTGAAGTCAA TAACAACATC CGTTTTGTTT GAATTGTTAT TGACTTCAAA TTATGCATAC ACTTAATAAA TGTTAAATTC ATTTTGAAAG CTATTTTTGT TTCATTATTT AAATCGAACA GGATGCCGAA ACGAAATATA TTATCATCAA TATAAAATGC GTGTCAATAA CAAACAAAAT ATACAATTTG CATATTAATA TTTCGAAATT GAACAAAAAA TTTTTAATAT AAATAGATCA CCTGAAAGCT GACATTTTAT TCAGTAAACA GTAAACACAA AAACGATCGC TTAATTCTTA TCATTTTTCT AAATTTCGAT TTTAAA*ATG*A AATTTGTGGT TGCTTTTATG GTTTTGGCCA T**TTGCAATCA Gg**CAATGACA GAATATTCTA TATGCCGTCA ATGCATTTCG AATTACGAAT CCTTCAAGTG ACCGTAGCGC CATCATTACA CAAGTACTAT TGAAAATTAT TCAGTGTTTC TCTGTTTGAG TGCGAATACA AACGTAACTC TCTCTGCGTC GCCTTTACCT CATGTTTCTA TTGTGCGACA CTTTGTGTAA TCGTGGCGCT GTAAGCAGTT GTCACATTCG AATACAATTC GACAGAATAT GGTGGCA**TTG CAATCAGGt**g aggaaaaaaa aaattataat aatattttac atttgatgat tgtcaaagtt ttgcgatata aaataatttt ggaaattatt tttttcggca gGCATTTGCC TCTCCACTTC CTCTGGCCTA TACTGATCAA AGTTATGATG CATGTGATCG AACATTTGAT GAAACGGGTC GAAATTGTCA ACCTTTATGT AATGCTATTT TCGGAAATCC GCTTGTATAT GAAAATCACG GCTCAGAGAC ATCATATGAA TGGAAACCAC CGCAACACAC TGAGACAGAG ACATTACATG AATGGAAACC ACCGCAACAC ACTGAGACAT CACATGAATG GAAACCACCG CAACACACTG AGACAGCCAA GAAGGAGAAG AAATCTAAAA AAAGAAAGCC AAA***TAA***TTAT TTCCAAAGTG ACATTCATTG TTTTGCATTG TAGTTCACAA TAAAATCGAG CATTTGACAA AACGGTAAAC AATTTGAAAA GAACGACGTG CAAAATGTAA AATTCTACCT ATATCCATCT CTTTTCTCAC TTAAAACCAA ATGAATTTCT TTCACATTGG TTCAATTTTG CACGTCGTTC CTTAAAAATT GCATTTTTAC TTAGAAAGTC AGAAATACAT ATTTGACGAG ATGAAAAAAA ACAAAACCAT TTATCTTCTT TCATTTTTAA ATTCTTAAGT TCACGGTCGT CAAATTTAAA CGTCTACTTT GCATTAATTG TGAATAAAAC GCAAAAAACA TTGAAAATTT TGTTTACATT CCATTCTTTT CATGAAATCG ATGCTGATGA ATATTCAAAT TTTATTTCTT TTGGGAAAAT ACTTAAATTG TCTCGAGCGC TTAAAAAAGC TTCTTCATCA CAATAGATCT GCGTTTATCG AAATTAATCA TCAGCCTCCA CTTGCATCGG CCAAGTGGAG CTAGCTTACT TTCTAAATCT AAACTTCCAA AGTGAATAAA CAGAAAAAAG CTTCAGTCGC CTGTTAAACT TAACTTAAAA AGCGTTTCAT CGATGAAAAG AGAAAAAATG TTGATTCCCT TGCTGATGAC A

C GTCAATAACA ACATCCGTTT TGTTTGAATT GTTATTGACT TCAAATTATG CATACACTTA ATAAATGTTA AATTCATTTT GAAAGCTATT TTTGTTTCAT TATTTAAATC GAACAGGATG CCGAAACGAA ATATATTATC ATCAATATAA AATGCGTGTC AATAACAAAC AAAATATACA ATTTGCATAT TAATATTTCG AAATTGAACA AAAAATTTTT AATATAAATA GATCACCTGA AAGCTGACAT TTTATTCAGT AAACAGTAAA CACAAAAACG ATCGCTTAAT TCTTATCATT TTTCTAAATT TCGATTTTAA A*ATG*AAATTT GTGGTTGCTT TTATGGTTTT GGCCATTTGC AATCAGgtga ggaaaaaaaa aaaaaattat aataatattt tacatttcat cattgtcaaa gttttgcgat ataaaataat tttggaaatt atttttttcg gcagGCATTT GCCTCTCCAC TTCCTCTGGC CTATACTGAT CAAGTTTATG ATGCATGTGA TCGACAATTT **GATGAAAC**CA TCGTATAGCA TCGTGAATAT TTTCACGTAT GTTGTTGCGC TACAGCGCCA CGTAATAGTA CCTTTTGGTA CTAATAGTGA TGTAACATGA CGGCGGTTAA AACGACTCAT TCAAAGTGAC GTTTCAAGCC GTTCACATTA TTTCACATCA GTTCACATTG TTTATGCCAG TTTATGGTTA GTGTTTTATT GAAAAATTTA ATCCCATTTC ACGAATTCCA TCGTTTGTTT CAATTTTTTT TTTATCAAGT GTTCTCAGTG ATGCTGAAAG ACAAAAATTA TGAATTGAAA TCCATAGAAT GATATTTTCG GTTATGCCAA ACAACTTAAA ACGACGGTTA TGTCAAAACA CGTGTTTTCA AAAGAGTATT ATAAGAGGGC GCTCTGCTAG AAGTGTCAAC GCGTGAGTTT CGAACAATTC ATACATTATC GTGACAAGTT GCTATGCCCT G**GATGAAAC**G GTTCGAAATT GTCAACCTTT ATGTAATGCT ATTTTCGGAA ATCCGCTTGT ATATGAAAAT CACGGCTCAG AGACATCATA TGAATGGAAA CCACCGCAAC ACACTGAGAC AGAGACATTA CATGAATGGA AACCACCGCA ACACACTGAG ACATCACATG AATGGAAACC ACCGCAACAC ACTGAGACAG CCAAGAAGGA GAAGAAATCT AAAAAAAAGA AAGCCAAA***TA A***TTATTTCCA AAGTGACATT CATTGTTTTG CATTGTAGTT CACAATAAAA TCGAGCATTT GACAAAACGG TAAACAATTT GAAAAGAACG ACGTGCAAAA TGTAAAATTC TACCTATATC CATCTCTTTT CTCACTTAAA ACCAAATGAA TTTCTTTCAC ATTGGTTCAA TTTTGCACGT CGTTCCTTAA AAATTGC
