# Supplementary material for: Avirulence Effector Discovery in a Plant Galling and Plant Parasitic Arthropod, the Hessian Fly (Mayetiola destructor)
Source: PLoS One. 2014 Jun 25;9(6):e100958. doi: 10.1371/journal.pone.0100958 (PMC4071006; doi:10.1371/journal.pone.0100958)
Supplement: Figure S4 — vH13 candidate gene 13 cDNA sequence. Purple lettering indicates one copy of a sequence that is followed by two imperfect copies. Underlined sequence corresponds to the dsRNA used to knockdown vH13 expression. (DOCX) [file pone.0100958.s004.docx]

ATTCAGTAAA CAGTAAACAC AAAAACAATC GCTTAATTCT TATCATTTTC CTGAATTTCG ATTTTAAAAT GAAATTTGTG GTTGCTTTTA TGGTTTTGGC CATTTGCAAT CAGGCATTTG CCTCTCCACT TCCTCTGGCC TATACTGATC AAGTTTATGA TGCATGTGAT CGACAATTTG ATGAAACGGT TCGAAATTGT CAACCTTTAT GTAATGCTAT TTTCGGAAAT CCGCTTGTAT ATGAAAATCA CGGCTCAGAG ACATCATATG AATGGAAACC ACCGCAACAC ACTGAGACAG AGACATCACA TGAATGGAAA CCACCGCAAC ACACTGAGAC ATCACATGAA TGGAAACCAC CGCAACACAC TGAGACAGCC AAGAAGGAGA AGAAATCTAA AAAAAAGAAA GCCAAATAAT TATTTCCAAA GTGACATTCA TTGTTTTGCA TTGTAGTTCA CAATAAAATC GAGCATTTGA CAAAAAA
