# Supplementary material for: Avirulence Effector Discovery in a Plant Galling and Plant Parasitic Arthropod, the Hessian Fly (Mayetiola destructor)
Source: PLoS One. 2014 Jun 25;9(6):e100958. doi: 10.1371/journal.pone.0100958 (PMC4071006; doi:10.1371/journal.pone.0100958)
Supplement: Figure S5 — Genomic DNA sequences of H13 -avirulent candidate-13 alleles. (A) Allele with three imperfect repeats. (B) Allele with two imperfect repeats. (C) Allele with one copy and no repeats. Colors and lettering are as described in Figure S2. (DOCX) [file pone.0100958.s005.docx]

A GTCAATAACA ACATCCGTTT TGTTTGAATT GTTATTGACT TCAAATTATG CATGATATTA TTTATTCTTA TTTTTTTTAT TACTTAATAA ATGTTAAATT CATTTTGAAA GCTATTTTTG TTTCATTATT TAAATCGAAC AGGATGCCGA AACGAAATAT ATTATCATCA ATATAAAATG CGTGTCAATA ACAAACAAAA TATACAATTT GCATATTAAT ATTTCGAAAT TGAACAAAAA TTTTTTAATA TAAATAGATC ACCTGAAAGC TGACATTTTA TTCAGTAAAC AGTAAACACA AAAACAATCG CTTAATTCTT ATCATTTTCC TGAATTTCGA TTTTAAA*ATG* AAATTTGTGG TTGCTTTTAT GGTTTTGGCC ATTTGCAATC AGgtgaggaa aaaaaaaaaa aattataata atattttaca tttgatgatt gtcaaagttt tgcgatataa aataattttg gaaattattt ttttcggcag GCATTTGCCT CTCCACTTCC TCTGGCCTAT ACTGATCAAG TTTATGATGC ATGTGATCGA CAATTTGATG AAACGGTTCG AAATTGTCAA CCTTTATGTA ATGCTATTTT CGGAAATCCG CTTGTATATG AAAATCACGG CTCAGAGACA TCATATGAAT GGAAACCACC GCAACACACT GAGACAGAGA CATCACATGA ATGGAAACCA CCGCAACACA CTGAGACATC ACATGAATGG AAACCACCGC AACACACTGA GACAGCCAAG AAGGAGAAGA AATCTAAAAA AAAGAAAGCC AAA***TAA***TTAT TTCCAAAGTG ACATTCATTG TTTTGCATTG TAGTTCACAA TAAAATCGAG CATTTGACAA AACGGTAGAC ATTTGAAAAG AACGACGTGC AAAATGTAAA ATTCTACCTA TATCCATCTC TTTTCTCACT TAAAACCAAA TGAATTTCTT TCACATTGGT TCAATTTTGC ACGTCGTTCC TTAAAAATTG C

B GTCAATAACA ACATCCGTTT TGTTTGAATT GTTATTGACT TCAAATTATG CATGATATTA TTTATTCTTA TTTTTTTTAT TACTTAATAA ATGTTAAATT CATTTTGAAA GCTATTTTTG TTTCATTATT TAAATCGAAC AGGATGCCGA AACGAAATAT ATTATCATCA ATATAAAATG CGTGTCAATA ACAAACAAAA TATACAATTT GCATATTAAT ATTTCGAAAT TGAACAAAAA TTTTTTAATA TAAATAGATC ACCTGAAAGC TGACATTTTA TTCAGTAAAC AGTAAACACA AAAACAATCG CTTAATTCTT ATCATTTTCC TGAATTTCGA TTTTAAA*ATG* AAATTTGTGG TTGCTTTTAT GGTTTTGGCC ATTTGCAATC AGgtgaggaa aaaaaaaaaa aattataata atattttaca tttgatgatt gtcaaagttt tgcgatataa aataattttg gaaattattt ttttcggcag GCATTTGCCT CTCCACTTCC TCTGGCCTAT ACTGATCAAG TTTATGATGC ATGTGATCGA CAATTTGATG AAACGGTTCG AAATTGTCAA CCTTTATGTA ATGCTATTTT CGGAAATCCG CTTGTATATG AAAATCACGG CTCAGAGACA TCATATGAAT GGAAACCACC GCAACACACT GAGACAGAGA CATCACATGA ATGGAAACCA CCGCAACACA CTGAGACAGC CAAGAAGGAG AAGAAATCTA AAAAAAAGAA AGCCAAA***TAA*** TTATTTCCAA AGTGACATTC ATTGTTTTGC ATTGTAGTTC ACAATAAAAT CGAGCATTTG ACAAAACGGT AGACATTTGA AAAGAACGAC GTGCAAAATG TAAAATTCTA CCTATATCCA TCTCTTTTCT CACTTAAAAC CAAATGAATT TCTTTCACAT TGGTTCAATT TTGCACGTCG TTCCTTAAAA ATTGC

C GTCAATAACA ACATCCGTTT TGTTTGAATT GTTATTGACT TCAAATTATG CATACACTTA ATAAATGTTA AATTCATTTT GAAAGCTATT TTTGTTTCAT TATTTAAATC GAACAGGATG CCGAAACGAA ATTATCATCA ATATAAAATT CGTGTCAATA ACAAACAAAA TATACAATTT GCATACTAAT ATTTCGAAAT TGAACAAAAA ATTTTTAATA TAAATAGATC ACCTGAAAGC TGACATTTTA TTCAGTAAAC AGTAAACACA AAAACGATCG CTTAATTCTT ATCATTTTTC TAAATTTCGA TTTTAAA*ATG* AAATTTGTGG TTGCTTTTAT GGTTTTGGCC ATTTGCAATC AGgtgaggaa aaaaaaaatt ataataatat tttacatttg atgattgtca aagttttgcg atataaaata attttggaaa ttattttttt cggcagGCAT TTGCCTCTCC ACTTCCTCTG GCCTATACTG ATCAAGTTTA TGATGCATGT GATCGACAAT TTGATGAAAC GGTTCGAAAT TGTCAACCTT TATGTAATGC TATTTTCGGA AATCCGCTTG TCTATGAAAA TCACGGCTCA GAGACATCAT ATGAATGGAA ACCACCGCAA CACACTGGAA CAGCCAAGAA GGAGAAGAAA TCTAAAAAAA AGAAAGCCAA A***TAA***TTATTT CCAAAGTGAC ATTCATTGTT TTGCATTGTA GTTCACAATA AAATCGAGCA TTTGACAAAA TAGTAAACAA TTTGAAAAGA ACGACGTGCA AAATGTAAAA TTTTACCTAT ATCCATCTCT TTTCTCACTT AAAACCAAAT GAATTTCTTC CTCAATTGGT TCAATTTTGC ACGTCGTTCC TTAAAAATTG C
