# Supplementary material for: Avirulence Effector Discovery in a Plant Galling and Plant Parasitic Arthropod, the Hessian Fly (Mayetiola destructor)
Source: PLoS One. 2014 Jun 25;9(6):e100958. doi: 10.1371/journal.pone.0100958 (PMC4071006; doi:10.1371/journal.pone.0100958)
Supplement: Table S1 — vH13 chromosome walk progression. (DOCX) [file pone.0100958.s006.docx]

**Table S1:** *vH13* chromosome walk progression.

| Step | BAC-end*** | Primers† | Postitive BACs (FP Contigs)‡ | Recomb.§ |
| --- | --- | --- | --- | --- |
| -1 | Mde11J6b | NS | Mde5J14 (512) | ND |
| 0 | STS-134 | gaattcacacgccgaacca ttaactagaaaaatgtgt | Mde11J6, Mde37L3 (56) | 6 |
| 1 | Mde11J6g | cagtttcgactatttactgc gcattaagcggtagttcgtt | Mde22L4, Mde1D20, Mde28J14, Mde32H7, Mde20H3, Mde22O10, Mde22M18 (56) | ND |
| 2 | Mde22L4b | tatcgacctgcaggcatgc ttgcaatacaaacacacac | Mde7P21, Mde46P20, Mde1E4, Mde10O20, Mde28E2, Mde34N24, Mde4E8 (56) | ND |
| 3 | Mde7p21b | ttgaaggaatttcttacacg tgtattcgagtaatgatacg | Mde41L6 (56) | ND |
| 4 | Mde41L6g | NS | Hf2A21, Hf2N10, Hf4N11, Hf12I23, Hf10K9 (12) | ND |
| 5 | Hf2A21b | gttgttagtgaagtgaatgg agtacgtttttccgttcacc | Hf6L22, Hf6J12 (12) | ND |
| 6 | Hf6L22g | aatcgagcagaattctgtgg acaacatcaacgaatttgcg | Hf14L18, Hf14P18, (12) | ND |
| 7 | Hf14L18g | acatatctcaaaagtgtcg ttatcgaacgctacacctg | Hf11F20, Hf9E12, Hf16P20, Hf16E4, Hf8E23, Mde21d18, Mde16C4 (12) | ND |
| 8 | Hf11F20b | aggatttttccgccattgc ggtaattacatgtaaatgg | Hf2P10, Hf16L19, Hf16A15, Hf2M17, Hf11A21, Hf11G24, Hf16J16, Hf14D2, Hf13E7 (12) | ND |
| 9 | Hf2P10 (SSR145) | tttcaaccacgaatcgaaca ttgcacggatagaaatcacg | Hf16K11, Hf16G14, Hf6G5, Hf5P5, Hf12L9, Hf9J21, CL21H11, CL17B9, CL31F15, CL26K13, CL29M22 (240) | 6 |
| 10 | Hf16K11 (SSR152) | tggaatcagccaattcttga ataatccgctcgacgaacaa | Hf5L20, Hf12C16, Hf12F4, Hf6C7, Hf12O13, Hf1E23, CL23N14, CL21G2 (421) | 6 |
| 11 | Hf5L20b | tgttatatgatttgacctgc acaatctacatatgaatgg | Hf3J13, Hf9N1, Hf9L6 | ND |
| 12 | Hf3J13g | gtgcaaaagacagagtttac tagaatactcaagctatgca | Hf16N3, Hf6N6, Hf11N24, Hf7F22, Hf6C24, Hf10M7, Hf4B10, Hf4J3, Hf4D16, Hf12P5, Hf10P16, Hf12P24, Hf12E1, Hf10D2, Hf12B2, Hf16E19 (827, 702) | ND |
| 13 | Hf16N3b | NS | CL27K24, Hf7L4, Hf1O18, Hf5D17, CL21F3 (702) | ND |
| 14 | CL27K24b | NS | CL18K13, Hf7L15, Hf8D16, CL27O24 (702) | ND |
| 14 | Hf7L15g | caacacacaatacaagacacac ttcctcgccaacaacaac | NP | 6 |
| 15 | CL18K13g | atttcggtcacacttacga attggcatttgcatcttt | Hf10B14, CL26A5 | 5 |
| 16 | Hf10B14g | cgaacaataggtgaaatcct cccgacgagacaaataaa | Hf5J21, Hf11J16, Hf4J11, Hf8J22, Hf11I17, CL19B6, CL23P4, CL23I18, CL27A6, CL32F13, CL32G13, CL30K21, Mde1E8, Mde24F9, Mde8F1, Mde11I22, Mde13D20, Mde23A3, Mde25B2, Mde26B18 | ND |
| 16 | Hf11j16b | tgtgtcagatgtttcagag aggcaagtgttggtctat | NP | 4 |
| 17 | Hf5J21b | ggctgtctttgagttttg gttcgtcttggtttctttc | Hf9P3 | ND |
| 18 | Hf9P3g | gctccaatcacaactctta atacactgaaccgaaacc | Hf11J21 | ND |
| 19 | Hf11J21g | cacaagcaaagagcaaagt aaagcaggacacaccatc | Hf8F11 | ND |
| 20 | Hf8F11b | ggctactggatgttgtcg cattgatttcggttgagc | Hf8K23 | 4 |
| 21 | Hf8K23b | ttgtcgcacctttcattc cgatagtctgattttgtttcg | Hf2L14, CL19N13, CL29A16, CL28C19, CL29D15, CL31G14, Hf3P20, Hf13N14, CL21A6, CL24I10, CL24H24 (1071 and 714) | 4 |
| 22 | Hf2L14b | gttcatctctatctccttcc atcttgcgtctgttactct | CL24B9, CL28F5, CL17I9, CL31J13, CL32G24, CL30M18 (912) | ND |
| 23 | CL24B9g | agcggaatcagtaatcgt gccaccagcattttattg | Hf4B18, Hf1E7, Hf5B12, Hf2D20 | ND |
| 23 | Hf1E7b | ccctgttattcaccatatac gattcatgttcagttgctat | NP | 4 |
| 24 | Hf4B18g | tgatgatgagaccaaagatg tatggctgacaagcaaatc | Hf2G3, Hf12O5, Hf4C23, CL17N6, CL21N14, CL29N3, CL27B20, CL29H23 (517) | 4 |
| 24 | Hf2G3b | gctcaaaccgaaatgtaaac aatggtcgtgatgcgtta | NP | 4 |
| 25 | Hf12O5g | cgtgtcccattcaaaatac gaaacatcaccaaaagcag | Hf1B2, Hf5C21, Hf8K15, Hf16D21, Hf8L24, CL21F6 | ND |
| 25 | Hf5C21b | gttcaatgtggggaaaac agagacgaccaaagacca | NP | 4 |
| 25 | Hf8K15b | attcgtggtcttcccttt atgcgtctattttctggtg | NP | 4 |
| 26 | Hf1B2g | attgttgtctgatggaagg cttgaccgtgtatttgg | CL56B18, CL72J23, Mde6B5, Mde7G11, Mde15L10, Mde22N22, Mde44N17, CL58P6, CL50G7, CL69P22, CL74L10, CL74P20, CL91E17, CL80I20 | 4 |
| 26 | CL72J23g | acaacgaatacctcaactg atggagagagagagaaagag | NP | 4 |
| 27 | CL56B18b | gatgttgatgtgttgtgag gtcattctctatctgtcttga | Hf1P2, Hf7L6, CL17H3, Hf4I15 | ND |
| 28 | Hf1P2g | gagatgaggaacaacaaag ccgaaagggaaaataagg | Hf3K6, Hf4O10, Hf6I4, CL20J5, CL19J7, CL23N15, CL18F21 | 3 |
| 29 | Hf3K6b | gttttgatgagacgaatgtg gttggtgatggtgtttgt | Hf12I20, Mde10I16 (1102) | ND |
| 30 | Hf12I20b | tctacgatacataaccgatg cacctcaatcaccttctc | Hf6J9, CL17A7, CL19M9, CL32I8, CL30C13, CL17F17, CL22A24, CL25K17 (322) | 3 |
| 31 | CL25O11b | gattcgttaatttgaatgac caatatgcatattaaatgcc | CL29N21, Hf13L4, Hf5P7, Hf3I13, Hf5M20, Hf2M8, Hf7H1 | ND |
| 31 | Hf13L4g | acagcagagtgggaatca ccttttatttggacttgtgg | NP | 1 |
| 32 | CL29N21b | cgggaaaagatgaaacaag catctcgtgccaatgaata | Hf3I13, Hf5P7, Hf5M20, Hf2M8, CL23I11 | 1 |
| 32 | CL29N21g | ttcatccattgcgtgtta gacgacataaagcgagtttc | NP | 0 |
| 32 | Hf5M20g | ctccacatagtccaaaatc gaagaaacagtaaacgagaac | NP | 0 |
| 32 | Hf3I13b | ccgtttgaagtgaagctaat cagtcaccgagaggtttg | NP | +1 |
| 32 | Hf5P7g | ccatttattgctttagctg ccatttattgctttagctg | NP | +1 |
| 33 | Hf3I13b | ttctcctttctcacttcg tactttggtatcggtcgta | Hf14O13, CL22B5 (880) | +1 |

***Markers were developed from BAC-end sequences and labeled “b” (T7-primed sequence) or “g” (m13-primed sequence), or from SSRs within the BACs. BAC CL25O11 did not hybridize in library screens but was present in FPC 322. †NS = no sequence available; PCR amplified the BAC-end using one primer complementary to either the T7 or M13 promotor and another primer complementary to a universal primer ligated to the nearest *Mse*I restriction site in the insert DNA. ‡FISH determined that underlined BACs were present on the X2 short arm. NP = hybridization not performed. §Number of individuals recombinant for the marker and *H13*-virulence and -avirulence in one mapping population (n=106); 1 and +1 were different recombinant individuals. ND = no data, not polymorphic, or recombination analysis was not performed.
