# Supplementary material for: Avirulence Effector Discovery in a Plant Galling and Plant Parasitic Arthropod, the Hessian Fly (Mayetiola destructor)
Source: PLoS One. 2014 Jun 25;9(6):e100958. doi: 10.1371/journal.pone.0100958 (PMC4071006; doi:10.1371/journal.pone.0100958)
Supplement: Table S2 — Predicted genes in the HF BAC Hf5p7 sequence. (DOCX) [file pone.0100958.s007.docx]

**Table S2:** Predicted genes in the HF BAC Hf5p7 sequence.

| Gene | Exons | AA | CDS | BLASTX* | Reference† | Putative protein |
| --- | --- | --- | --- | --- | --- | --- |
| 1 | 4 | 249 | (+) 5566-7937 | 2e-80 | Cq- XP_001850654.1 | short-chain dehydrogenase |
| 2 | 2 | 177 | (+) 11456-12152 | 5e-28 | Ag- XP_309789.3 | short-chain dehydrogenase |
| 3 | 2 | 81 | (-) 13075-12632 | NSS |  |  |
| 4 | 2 | 331 | (-) 17494-15359 | 9e-11 | Aa- XP_001651665.1 | cuticular protein |
| 5 | 2 | 250 | (+) 19144-20002 | 5e-17 | Ag- XP_001230655.1 | cuticular protein |
| 6 | 4 | 309 | (+) 22151-24314 | 1e-73 | Ag- XP_315532.3 | short-chain dehydrogenase |
| 7 | 3 | 155 | (-) 40769-32873 | 9e-21 | Dm- NP_001097395.1 | Rgk3 |
| 8 | 1 | 811 | (+) 43588-46023 | 1e-95 | Ap- XP_001942753.1\| | non-LTR retrovirus reverse transcriptase |
| 9 | 2 | 78 | (-) 47936-47378 | 9e-10 | Ag- XP_314147.4 | Ras-like GTPase |
| 10 | 3 | 128 | (+) 71145-75283 | NSS |  |  |
| 11 | 8 | 886 | (-) 83857-75955 | 0.0 | Aa- XP_001664102.1\| | cAMP 3,5-cyclic phosphodiesterase |
| 12 | 4 | 262 | (-) 112431-105008 | NSS |  |  |
| 13 | 2 | 90 | (+) 117122-117488 | NSS |  |  |
| 14 | 3 | 157 | (+) 118390-124426 | NSS |  |  |

*NSS= no significant similarities. †Aa= *Aedes aegypti*, Ag= *Anopheles gambiae*, Ap= *Acyrthosiphon pisum* (pea aphid), Cq= *Culex quinquefaciatus*, Dm= *Drosophila melanogaster*.
