# Supplementary material for: Avirulence Effector Discovery in a Plant Galling and Plant Parasitic Arthropod, the Hessian Fly (Mayetiola destructor)
Source: PLoS One. 2014 Jun 25;9(6):e100958. doi: 10.1371/journal.pone.0100958 (PMC4071006; doi:10.1371/journal.pone.0100958)
Supplement: Table S3 — Marker and primer positions in the HF BAC Hf5p7 sequence. (DOCX) [file pone.0100958.s008.docx]

**Table S3:** Marker positions in the HF BAC Hf5p7 sequence.

| Marker | Position (bp) | Forward primer | Reverse primer |
| --- | --- | --- | --- |
| a | 1,832-1,987 | CCCAACAAAGAAAGACACAT | CATCTCGTGCCAATGAATA |
| b | 27,867-28,172 | ATGTTCAAAGCAGGAGGT | CCTACACCAATCTCATCAAT |
| c | 47,979-48,201 | CGGTTTCTATCTATTTCCTT | ATTCATCTGGTGTGTGTATT |
| d | 72,873-73,128 | GCTCAACACAAAAACACG | CATACCAAAACACATTCCTG |
| e | 83,296-83,561 | TACAAGAGAGACTGGAGAGC | ACACACTGAACACGCTTT |
| f | 99,784-100,144 | ATTCAAAAGCGAAAGTGCTA | AGCGGTAAATGACAAAACTC |
| g | 110,386-110,628 | GAAATCCATAGCATAATCG | GACCAAAATGACAACATACA |
| h | 116,700-118,134 | GCATCGCAAACAAAAGCAA | TGTCATCAGCAAGGGAATCA |
| i | 133,428-133,625 | CCATTTATTGCTTTAGCTG | CAAACAACATGAAACTGC |
